# The influence of multiple episodes of acute kidney injury on survival and progression to end stage kidney disease in patients with chronic kidney disease

**DOI:** 10.1371/journal.pone.0219828

**Published:** 2019-07-18

**Authors:** Lynne Sykes, Ozgur Asar, James Ritchie, Maharajan Raman, Diana Vassallo, Helen V. Alderson, Donal J. O’Donoghue, Darren Green, Peter J. Diggle, Philip A. Kalra

**Affiliations:** 1 Department of Renal Medicine, Salford Royal NHS Foundation Trust, Salford, United Kingdom; 2 Department of Biostatistics and Medical Informatics, Acibadem Mehmet Ali Aydinlar University, Istanbul, Turkey; 3 CHICAS Research Group, Lancaster Medical School, Lancaster University, Lancaster, United Kingdom; Kaohsiung Medical University Hospital, TAIWAN

## Abstract

**Background:**

Acute kidney injury (AKI) and chronic kidney disease (CKD) are common syndromes associated with significant morbidity, mortality and cost. The extent to which repeated AKI episodes may cumulatively affect the rate of progression of all-cause CKD has not previously been investigated. In this study, we explored the hypothesis that repeated episodes of AKI increase the rate of renal functional deterioration loss in patients recruited to a large, all-cause CKD cohort.

**Methods:**

Patients from the Salford Kidney Study (SKS) were considered. Application of KDIGO criteria to all available laboratory measurements of renal function identified episodes of AKI. A competing risks model was specified for four survival events: Stage 1 AKI; stage 2 or 3 AKI; dialysis initiation or transplant before AKI event; death before AKI event. The model was adjusted for patient age, gender, smoking status, alcohol intake, diabetic status, cardiovascular co-morbidities, and primary renal disease. Analyses were performed for patients’ first, second, and third or more AKI episodes.

**Results:**

A total of 48,338 creatinine measurements were available for 2287 patients (median 13 measures per patient [IQR 6–26]). There was a median age of 66.8years, median eGFR of 28.4 and 31.6% had type 1 or 2 diabetes. Six hundred and forty three (28.1%) patients suffered one or more AKI events; 1000 AKI events (58% AKI 1) in total were observed over a median follow-up of 2.6 years [IQR 1.1–3.2]. In patients who suffered an AKI, a second AKI was more likely to be a stage 2 or 3 AKI than stage 1 [HR 2.04, p 0.01]. AKI events were associated with progression to RRT, with multiple episodes of AKI progressively increasing likelihood of progression to RRT [HR 14.4 after 1 episode of AKI, HR 28.4 after 2 episodes of AKI]. However, suffering one or more AKI events was not associated with an increased risk of mortality.

**Conclusions:**

AKI events are associated with more rapid CKD deterioration as hypothesised, and also with a greater severity of subsequent AKI. However, our study did not find an association of AKI with increased mortality risk in this CKD cohort.

## Introduction

Acute kidney injury (AKI) and chronic kidney disease (CKD) are highly prevalent syndromes that are based on the same spectrum. Both AKI and CKD are associated with significant morbidity, mortality and Healthcare cost.[1–3] AKI has an incidence of 12–18% in recent United Kingdom (UK) studies of hospitalized patients.[4,5] Occurrence was associated with subsequent longer in-patient stays, and with over 40,000 deaths per annum.[4] The cost of treating AKI is estimated at 1% of the annual NHS budget, more than £1 billion annually.[4]

CKD affects 8–11% of the adult population, and is increased in populations with metabolic or cardiovascular co-morbidities. Whilst some patients do have predictable loss of glomerular filtration rate (GFR) over time, rates of CKD progression are variable and often non-linear.[6,7] Several studies already report the relationship between recurrent AKI episodes and CKD.[8–10] Hence CKD as a risk factor for the development of AKI is well established.[5,11] Recent data suggest that this may be a circular relationship.[12] Indeed, non-recovery from AKI may be the precipitant to CKD.[11–13] Furthermore, episodes of AKI in CKD patients are associated with more rapid transition between stages of CKD and increased risk for progression to end stage renal disease (ESRD) and the need for renal replacement therapy (RRT).[14,15]

What requires further evaluation is the extent to which repeated AKI episodes have a cumulative effect on worsening CKD prognosis. Likewise, further research is required into whether the severity of AKI events influences the long-term CKD outcome.[16,17] We explored these theories in this study, alongside secondary aims such as determining whether there is a difference in likelihood of CKD progression after AKI between different primary renal diseases.

## Methods

### Patient population

This analysis was performed as part of the Salford Kidney Study (SKS). This is a prospective study of patients referred to a single, secondary care renal centre for management of CKD. The centre serves a catchment population of 1.5 million people. All referred patients with CKD aged over 18 years and with capacity to provide informed consent are eligible for recruitment. In the non-dialysis cohort, patients who are expected to progress to RRT within six months of recruitment are not approached. All patients provide written informed consent. The study complies with the declaration of Helsinki and local ethical approval was obtained (current REC reference 15/NW/0818, North West—Greater Manchester South Research Ethics Committee). Patients selected for this analysis were those recruited between the study start date, 15^th^ November 2000, and 28^th^ February 2013.

### Study protocol and data collection

The design of the non-dialysis component of the SKS has been described previously under its previous title of the Chronic Renal insufficiency Standards Implementation Study (CRISIS).[18] In brief, demographic and clinical information are obtained at baseline and thereafter on an annual basis by means of a structured patient questionnaire delivered by trained research nurses. Reported co-morbidities and health issues are validated by reference to clinical notes stored within the local electronic patient record (EPR), through communication with their primary care provider, or by other secondary care providers in cases where admission to outlying hospitals occurred. All patients have a standardised biochemical and haematological analysis performed on an annual basis. Additional laboratory data collected as part of routine clinical care are also available for analysis. The integrated informatics and laboratory systems mean that this collection of additional laboratory data included those specimens collected by other local healthcare providers, including in Primary Care. The results of this study therefore include data relating to community acquired and managed AKI as well as those managed in hospital.

The key collected data included in the model were: 1) demographic (age, gender, height, weight, ethnicity, postcode); 2) renal (primary cause of CKD); 3) co-morbid conditions (diabetes mellitus, major cardiovascular events, smoking history, alcohol intake); 4) biochemical (serum creatinine [all values occurring over follow up were available for download from the EPR]; estimated glomerular filtration rate [eGFR] calculated using the 4-variable MDRD equation concomitant with creatinine measurement, urine protein:creatinine ratio). Pre-defined end-points were: death or initiation of chronic RRT (defined as the date of first session of chronic haemodialysis or peritoneal dialysis, or date of renal transplantation).

### Definition of AKI

Episodes of AKI were retrospectively identified in the SKS non-dialysis CKD population according to the KDIGO definition of AKI, by analysis of all repeated measurements of serum creatinine in each patient. Measurement of urine output for the extended KDIGO criteria was not available for consideration.[19] Patients who suffered AKI 3 could be captured in SKS because acute renal organ support is a defined end point within the study.

The relative change (RC) in serum creatinine (SCr) between two successive measurements (t and s) is calculated according to (SCr_t_−SCr_s_)/(SCr_s_). The classification of AKI events are as follows

- Stage 1: 0.5 ⩽ RC < 1 or an absolute increase in SCr ≥ 26.5 μmol/L;
- Stage 2: 1⩽ RC < 2

Stage 3: RC > 2 or SCr ≥ 353.6 μmol/L or initiation of renal replacement therapy

In cases where multiple flags occurred within 7 days, the attributed grade of AKI was determined by the nadir of renal function (i.e. the greatest increase in creatinine) during this period. Patients with serum creatinine measurements taken during dialysis had those values excluded. A team of three independent nephrology research fellows (RR, DV, HVA) reviewed all potential AKI episodes. Where there was disagreement, adjudication was performed by PK and JR.

### Statistical methodology

Up to four time periods were considered for each patient in this analysis. These were: time from recruitment to first AKI event; time from first to second AKI event; time from second to third AKI event; time after third AKI event.

Based on previous work[20] considering the influence of a single AKI episode on rate of eGFR loss, a linear mixed effects model was specified to calculate eGFR trajectory. A random intercept and serially correlated random effect were included in the model to account for deviations from the population-averaged intercept and population-averaged slope at a given time. This was specified as a zero-mean Gaussian stochastic process with exponential correlation function. Parameters were estimated using maximum likelihood.[21]

The Generalised estimating equation (GEE) method was originally proposed for applications in which a large number of subjects all experienced the same small set of follow-up times. In these circumstances, the covariance structure of the repeated measurements on each subject could be estimated non-parametrically without much loss of precision and with a gain in robustness.

The linear mixed model, in contrast, specifies the covariance structure of a sequence of repeated measurements at any set of follow-up times using a parsimonious model whose parameters can be estimated efficiently by the maximum likelihood method.

In the current setting, the follow-up times vary very substantially between patients and include some very long sequences. Non-parametric estimates of the covariance structure of repeated measurements would therefore be very unreliable and we prefer to use the more efficient linear mixed model approach.

As both death and progression to RRT are competing risks for further AKI episodes, competing risks models, a cause-specific Cox-model, were specified at each time point. These models considered four survival events: AKI 1; AKI 2/3; RRT before *n*th AKI event; Death before *n*th AKI event.

In the competing risks models, parameters were estimated by partial likelihood.[22] Cumulative incidence plots were used to graphically explore survival, as these remain unbiased where there are more than two outcomes.

## Results

### Study population

There were 2287 patients recruited to the SKS at the time of analysis. There were 48,338 repeated measurements of eGFR, of which 42,861 (88.7%) were outpatient readings. A total of 9262 eGFR readings (19.2%) were excluded from calculations of eGFR slope due to their measurement being in the immediate peri-AKI period as defined in the methods section. The number of repeated measurements per patients ranged between 1 and 280, with a median of 13.

The majority of patients were Caucasian (96.2%), with a predominance of males (62%), 67% of patients were current or ex-smokers, 48% drank alcohol, and 20% had suffered a previous cardiovascular event. Age at recruitment ranged between 20.0 and 94.3 years (median = 66.8 years) with a mean baseline eGFR of 30.5 ml/min/1.73m^2^. The most commonly coded cause of CKD was vascular (hypertension or renovascular disease; 24.6%), followed by glomerulonephritis (16.8%) and then diabetic renal disease (16.4%). Less common primary diseases were pyelonephritis (6.0%) and autosomal dominant polycystic kidney disease (ADPKD; 5.7%). These demographic details are shown in Table 1.

**Table 1:** Demographics of patient cohort.

Variable	Category/Statistics	Frequency (%)/Values
Number of patients		2287
Total number of measurements		48,338
Location where blood sample taken	Inpatient	5477 (11.3%)
	Outpatient	42,861 (88.7%)
Gender	Female	873 (38.2%)
	Male	1414 (61.8%)
Ethnicity	Caucasian	2200 (96.2%)
	Other	87 (3.8%)
Base hospital (at study entry)	Salford Royal Foundation Trust	681 (29.8%)
	Other	1606 (70.2%)
Smoking	Never smoked	749 (32.7%)
	Ex/current	1538 (67.3%)
Alcohol consumption	No	1191 (52.1%)
	Yes (any intake)	1096 (47.9%)
Diabetes	No	1565 (68.4%)
	Type I or II	722 (31.6%)
Previous major cardiovascular events	No	1832 (80.1%)
	Yes	455 (19.9%)
Primary renal disease	Diabetes	375 (16.4%)
	Glomerulonephritis	383 (16.8%)
	Immune/vasculitis	64 (2.8%)
	Polycystic	131 (5.7%)
	Pyelonephritis	137 (6.0%)
	Vascular	635 (27.8%)
	Other	562 (24.6%)
Baseline age (years)	Min	20
	50th quantile (Median)	66.8
	Max	94.3
Number of repeated measurements	Min	1
	50th quantile (Median)	13
	Max	280
Total follow-up (years elapsed between first and last measurements)	Min	0
	50th quantile (Median)	2.6
	Max	10.9
Baseline eGFR	Min	2.6
	50th quantile (Median)	28.4
	Max	116.5
Censored		1360 (59.5%)
Renal replacement therapy		356 (15.6%)
Death (before RRT)		571 (30.0%)

Several factors had a statistically significant association with baseline renal function. These included geographical variation, and variation between primary renal diseases. Vasculitis was associated with the highest baseline function (+4.43 ml/min/1.73m^2^ compared to the population mean). Higher baseline kidney function was also seen in patients who reported alcohol consumption (+6.7%, +1.5 ml/min/1.73m^2^). Lower baseline kidney function was associated with increasing age. For each year increase in age, mean kidney function decreased by 0.4% per year (-0.1 ml/min/1.73m^2^). Lower levels of baseline renal function were seen in patients with diabetic renal disease (-19%, -5.9 ml/min/1.73m^2^), ADPKD (-23%, -6.1 ml/min/1.73m^2^), and chronic pyelonephritis (1%, -3.7 ml/min/1.73m^2^).

**Fig 1:**
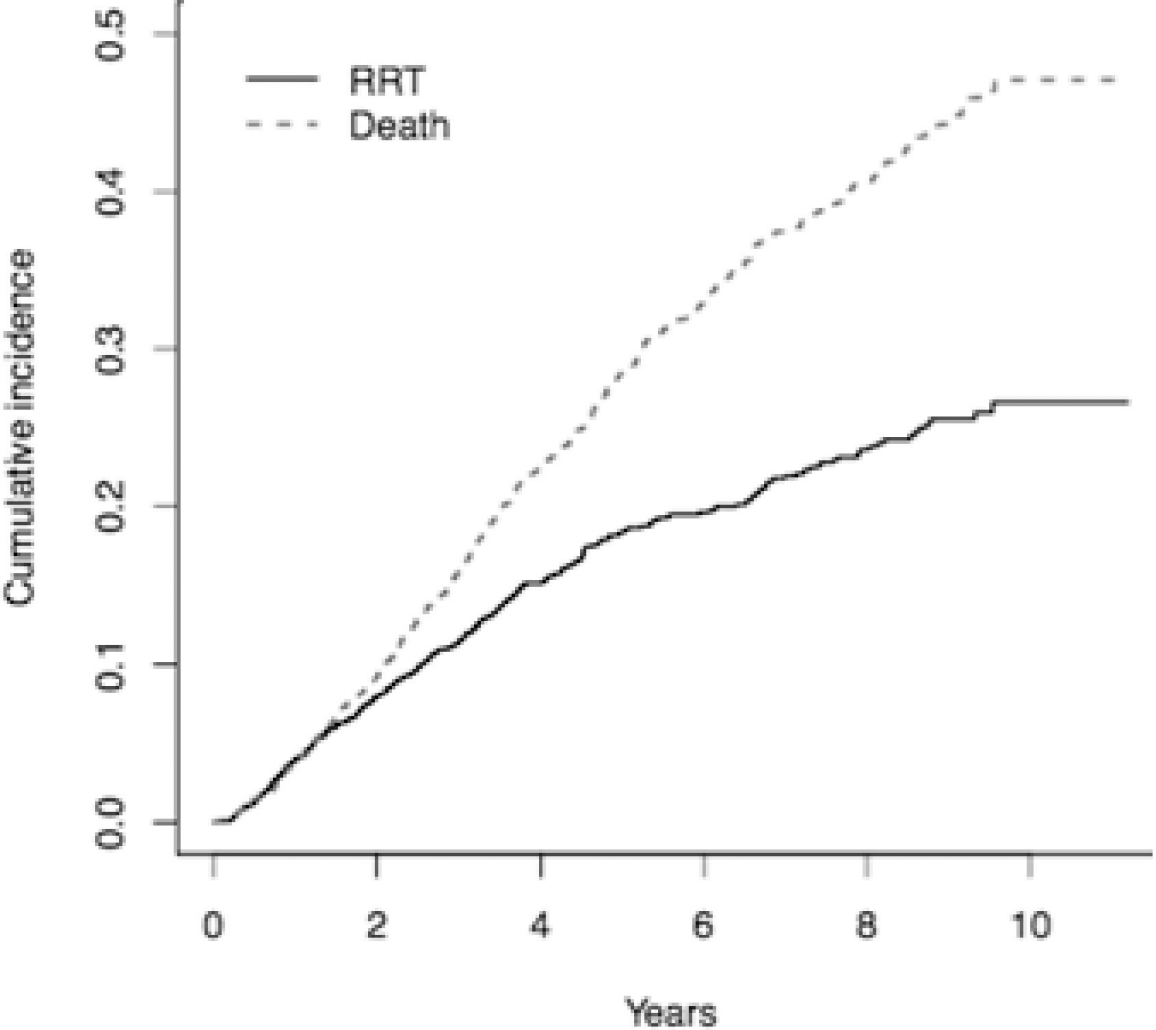
Cumulative incidence of outcome in the study population. RRT = renal replacement therapy.

Patient follow-up time ranged between 0 (i.e. only one creatinine measurement) and 10.9 years (median 2.56 years, IQR 1.09–4.60 years). 332 patients (14.5%) progressed to RRT, and 534 (23.3%) died prior to initiation of RRT. The incidence of RRT decreased over time, whilst incidence of death appeared constant (Fig 1). Administrative censoring occurred in 1338 cases (58.5%).

### AKI episodes

**Fig 2:**
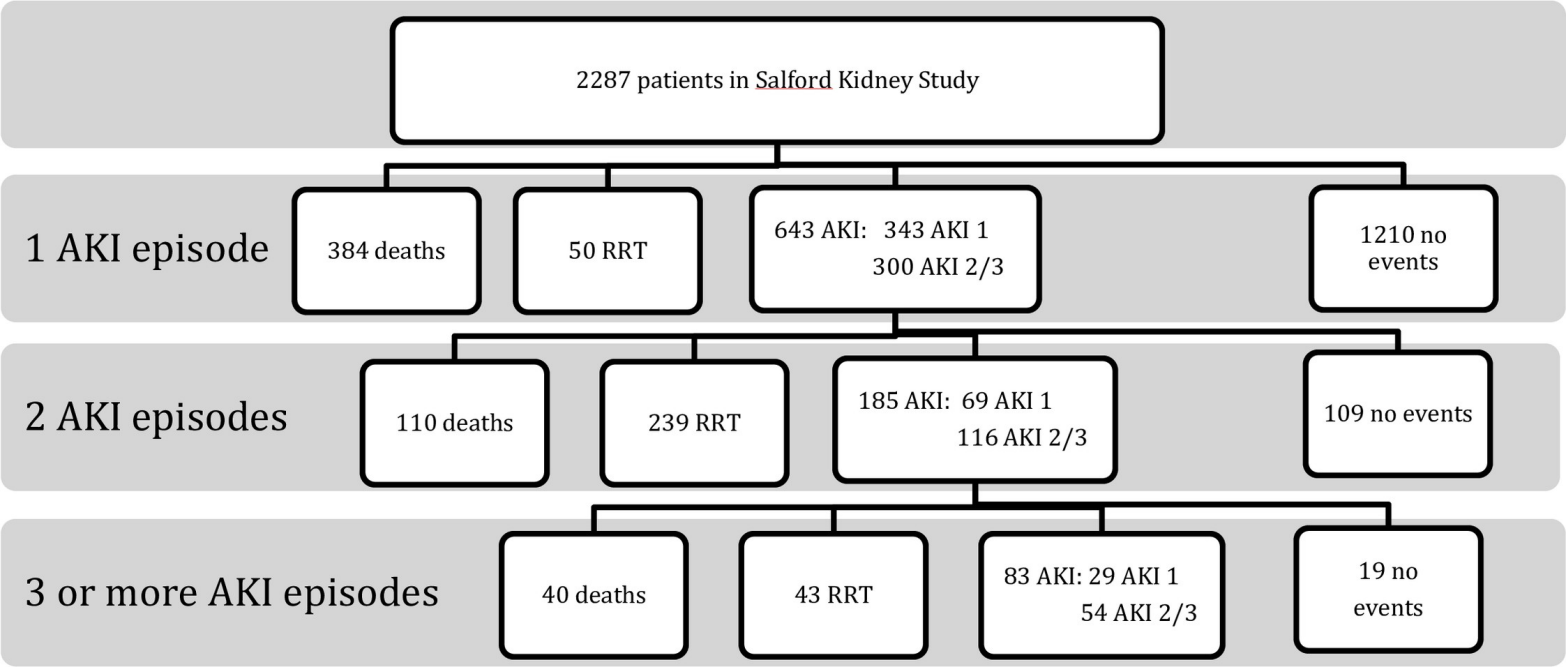
Consort diagram to show outcomes.

In total, 1000 AKI events were observed within this dataset. The majority were AKI 1 (n = 523, 52.3%), with 47.7% being AKI 2 or 3 (n = 477). At least one AKI event was observed in 643 patients (28.1%). Of the first AKI events, 343 were AKI 1 (53.3%) and 300 were AKI 2 or 3 (46.7%). With each additional AKI episode, an increasing proportion were numerically more severe AKI (i.e. stage 2 or 3 rather than AKI 1) compared to previous AKI events. Two or more AKI episodes were observed in 185 patients (8.1%). Of these second AKI events, 69 were AKI 1 (37.2%), and 116 were AKI 2 or 3 (62.7%, compared with 46.7% of first AKI as detailed above). Three or more AKI episodes were observed in 83 patients (3.6%). Of these third or more AKI events, 29 were AKI 1 (34.9%), and 54 AKI 2 or 3 (65.1%). This is shown in the consort diagram in Fig 2.

### Survival analysis, first AKI event

**Fig 3:**
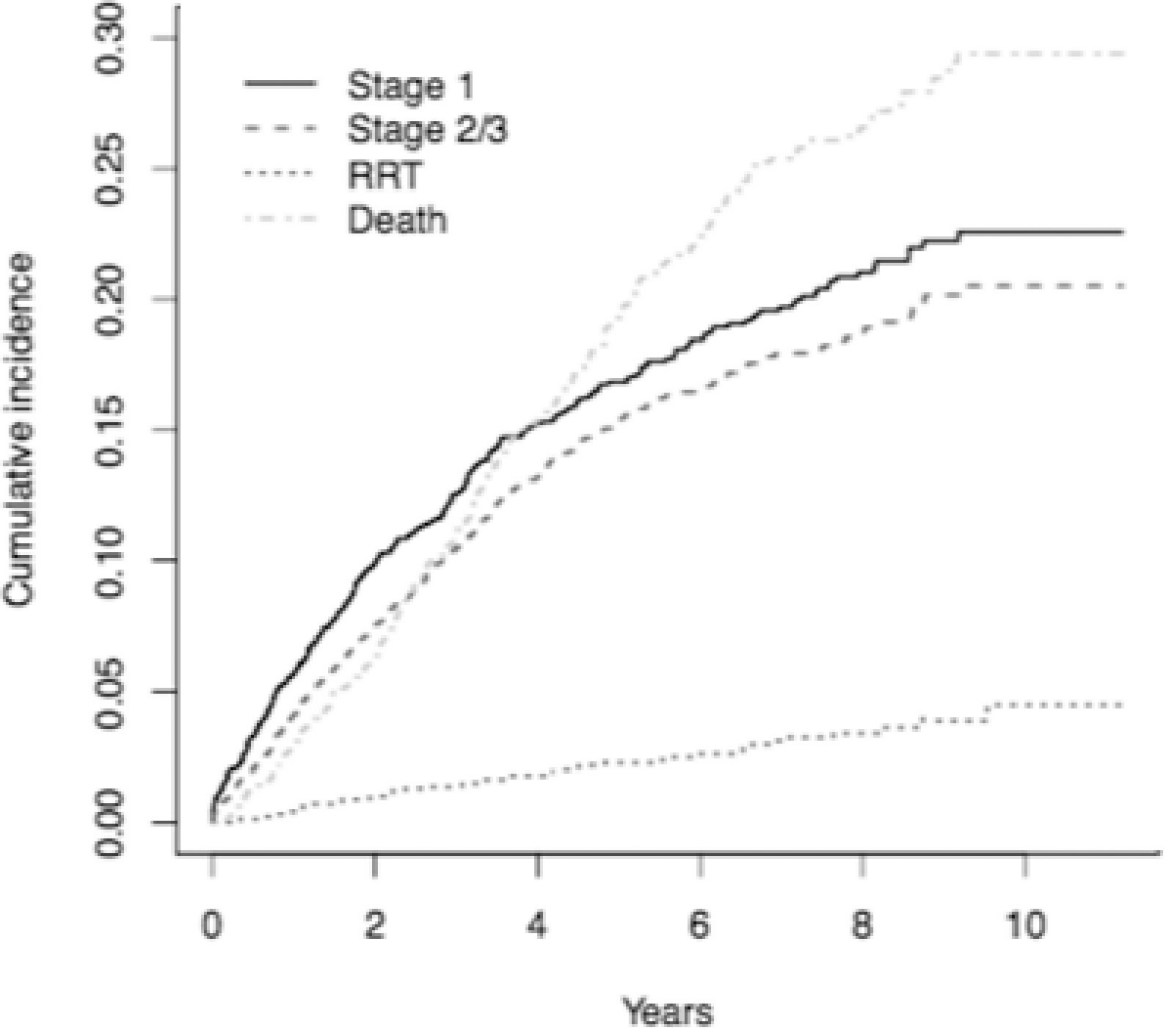
Cumulative incidences of first study end points in the SKS population. RRT = renal replacement therapy. “Stage 1” and “Stage 2/3” refer to first AKI event.

50 patients (2.2%) progressed to chronic RRT prior to the first AKI event, and 384 (16.8%) died before suffering an AKI event (Fig 3). 643 patients went on to have one or more AKI events during follow up; 300 (13.1%) had an AKI stage 2 or 3, and 343 (15.0%) had a stage 1 AKI stage 1 but no stage 2 or 3 events. No outcome events were observed in 1210 patients (52.9%). These were administratively censored.

Clinical factors associated with first event being AKI 1 were a smoking history (HR1.38; 95% CI 1.08–1.77, p = 0.01), diabetic renal disease (HR 1.55; 95% CI 1.04–2.31, p = 0.031) and autoimmune or vasculitic renal disease (HR 1.90; 95% CI 1.14–3.18, p = 0.014). An increased risk for AKI stage 2 or 3 was associated with ADPKD (HR 2.23; 95% CI 1.46–3.39, p<0.001) and older age (HR 1.01 per year; 95% CI 1.01–1.02, p = 0.015). Summary results for likelihood ratios for a first AKI event are presented in Table 2 with complete results in Table A in S1 File.

**Table 2:** Summary of significant variables for stage of AKI.

Variable	HR (95% CI)	p-value
**Significant risk for stage of first AKI**		
**Stage 1 AKI**		
Base hospital (tertiary centre)	2.31 (1.86, 2.86)	<0.001
Ex or current smoker	1.38 (1.08, 1.77)	0.01
Primary renal disease diabetes	1.55 (1.04, 2.31)	0.03
Primary renal disease immune or vasculitis	1.90 (1.14, 3.18)	0.01
**Stage 2/3 AKI**		
Baseline age (years)	0.99 (0.98, 1.00)	0.02
Primary renal disease polycystic kidneys	2.23 (1.46, 3.39)	<0.001
**Significant risk for stage of second AKI**		
**Stage 1 AKI**		
First AKI (stage 2/3)	0.49 (0.95, 0.85)	0.01
Ex or current smoker	0.65 (0.43, 1.00)	0.05
**Stage 2/3 AKI**		
Nil significantly associated		
**Significant risk for stage of third or more AKI**		
**Stage 1 AKI**		
Nil significantly associated		
**Stage 2/3 AKI**		
Nil significantly associated		

### Survival analysis, second AKI event

**Fig 4:**
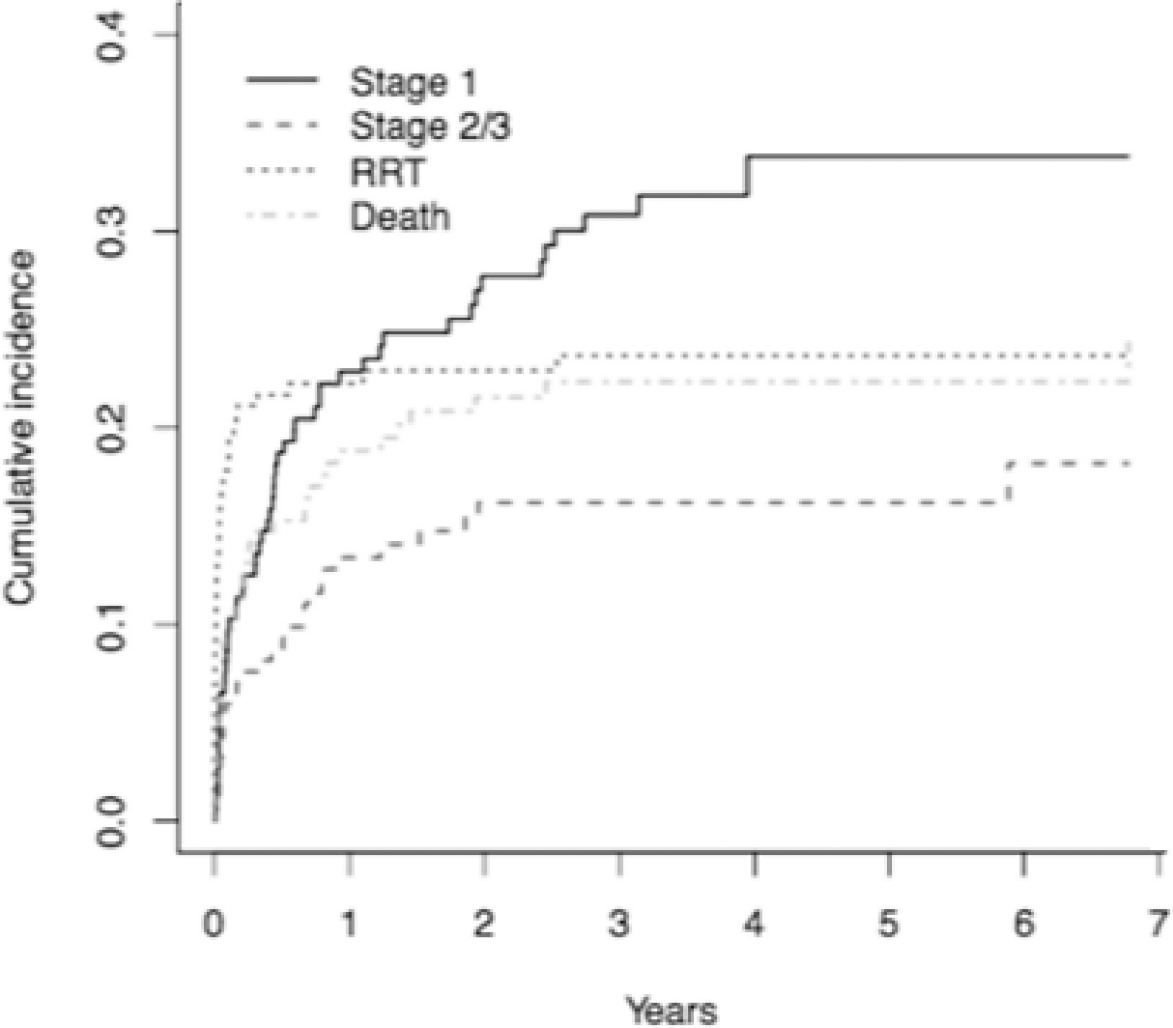
Cumulative incidences of study end points after any second acute kidney injury. RRT = renal replacement therapy. “Stage 1” and “Stage 2/3” refer to first AKI event.

Of the 643 patients who suffered a first AKI event, 185 (28.8%) then had a second AKI; 69 cases were AKI 1 (37.2%), and 116 were AKI 2 or 3 (62.7%, compared with 46.7% of first AKI as detailed above). There were 239 patients (37.2%) who progressed to chronic RRT before a second AKI occurred, and 110 (17.1%) died before a second AKI A time dependent comparison of these events is shown in Fig 4. In 109 patients (16.9%) patients no further events occurred and were therefore administratively censored.

In the 185 patients who suffered a second AKI episode, patients who had suffered an AKI stage 2 or 3 (in comparison to an AKI 1) as their first event had a greater risk for their second event also being an AKI 2 or 3 (HR 2.04; 95% CI 1.18–3.45, p = 0.011). No other clinical characteristics were significantly associated with risk for second AKI being stage 2 or 3 compared to stage 1. Summary results of likelihood ratios for factors associated with a second AKI event are presented in Table 2 with complete results in Table B in S1 File.

### Survival analysis, three or more AKI events

Of the 185 patients who had suffered two AKI episodes, 83 (44.9%) of these patients went on to have three or more episodes of AKI. 29 (15.7%) had a further AKI stage 1, 54 (29.2%) had a further AKI stage 2 or 3. 43 patients (23.2%) progressed to chronic RRT before a third AKI event, and 40 (21.6%) died before a third AKI event. There were no statistically significant characteristics associated with the risk for a third AKI event of any stage. Summary results of likelihood ratios for factors associated with a third AKI event are presented in Table 2 with complete results in Table C in S1 File.

### Survival analysis, mortality

Baseline factors significantly associated with risk for death prior to the first AKI episode included increased age (HR 1.08 [1.07–1.09] per year, p<0.001), male gender (HR 1.27 [1.01–1.59], p = 0.039), smoking (HR 1.39 [1.09–1.77], p = 0.008), diabetes mellitus (HR 1.58 [1.23–2.03], p = <0.001) and pre-existing cardiovascular disease (HR 1.36 [1.09–1.69], p = 0.007). Alcohol consumption was associated with a lower risk of death (HR 0.77 [0.62–0.95], p = 0.017). A summary is presented in Table 3 with the full model found in Table D in S1 File.

**Table 3:** Summary of significant variables for risk of death.

Variable	HR (95% CI)	p-value
**Significant risk for death prior to first AKI**		
Baseline age (years)	1.08 (1.07, 1.09)	<0.001
Base hospital (tertiary centre)	0.60 (0.48, 0.72)	<0.001
Male gender	1.27 (1.01, 1.59)	0.04
Ex or current smoker	1.39 (1.09, 1.77)	0.01
Any alcohol intake	0.77 (0.62, 0.95)	0.02
Diabetes (type I/II)	1.58 (1.23, 2.03)	<0.001
Major cardiovascular event	1.36 (1.09, 1.70)	0.01
**Significant risk for death prior to second AKI**		
Baseline age (years)	1.05 (1.03, 1.07)	<0.001
Major cardiovascular event	1.52 (1.00, 2.32)	0.05
Primary renal disease diabetes	2.14 (0.99, 4.62)	0.05
Primary renal disease immune or vasculitis	3.41 (1.29, 9.03)	0.01
Primary renal disease vascular	2.10 (1.12, 3.61)	0.02
**Significant risk for death prior to third or more AKI**		
Baseline age (years)	1.06 (1.02, 1.10)	<0.001
Any alcohol intake	2.28 (1.08, 4.84)	0.03
Major cardiovascular event	2.80 (1.27, 6.20)	0.01
Primary renal disease glomerulonephritis	3.56 (1.10, 11.54)	0.03

Following the first AKI episode and prior to the second AKI, only patient age remained significantly associated with risk for death (HR 1.053 [1.033–1.073], p = <0.001). An increased risk for death was associated with immune mediated (HR 3.411 [1.288–9.034], p = 0.014) and vascular causes of kidney disease (HR 2.009 [1.119–3.609], p = 0.020). A summary is found in Table 3 with the full model output found in Table E in S1 File. For patients who survived following a second episode of AKI, age (HR 1.059 [1.021–1.097], p = 0.002), alcohol (HR 2.284 [1.078–4.840], p = 0.031), cardiovascular disease (HR 2.800 [1.265–6.200], p = 0.011), and glomerulonephritis as primary disease (HR 3.564 [1.101–11.541], p = 0.034) were significantly associated with risk for death. A summary is presented in Table 3 with the full model output found in Table F in S1 File.

### Survival analysis, renal replacement therapy

In the period after a first AKI episode, those patients whose first episode of AKI was stage 2 or 3 had a fourteen-fold increased risk for RRT during follow up compared to patients who had suffered an AKI 1 (HR 14.46 [9.56–21.87], p <0.001). In the period after a second AKI episode (n = 185), those patients whose second AKI had been stage 2 or 3 had a twenty-eight fold increase in risk for progression to RRT compared to AKI stage 1 patients (HR 28.39 [9.71–82.99], p <0.001). A summary of complete results for all model outputs for RRT as outcome for these two time periods, and for prior to any AKI, are shown in Table 4 with complete results in the Tables G, H and I in S1 File.

**Table 4:** Summary of significant variables for risk of RRT.

Variable	HR (95% CI)	p-value
**Significant risk for RRT prior to first AKI**		
Baseline age (years)	0.97 (0.95, 0.99)	0.01
Base hospital (tertiary centre)	0.45 (0.21, 0.96)	0.04
**Significant risk for RRT prior to second AKI**		
First AKI (stage 2/3)	14.46 (9.56, 21.87)	<0.001
Baseline age (years)	0.99 (0.98, 1.00)	0.01
Base hospital (tertiary centre)	0.44 (0.31 0.62)	<0.001
Primary renal disease polycystic kidneys	2.11 (1.33, 3.33)	<0.001
Primary renal disease pyelonephritis	2.29 (1.28, 4.07)	0.01
**Significant risk for RRT prior to third or more AKI**		
Second AKI (stage 2/3)	28.39 (9.71, 83.00)	<0.001
Base hospital (tertiary centre)	0.34 (0.16, 0.73)	0.01
Diabetes (type I/II)	0.22 (0.06, 0.89)	0.03

## Discussion

This study presents several findings relevant to the prognosis of patients with chronic kidney disease who suffer acute kidney injury. We have confirmed the findings of previous studies that have shown patient age to be a risk factor for the development of AKI.[13] Our findings add to this by suggesting that age could be considered as a dynamic risk factor relevant to stage of AKI. Given that many risk stratification tools for AKI define age greater than 65 years[23] as a risk factor, it may be that this affords sensitivity to AKI 1 at the risk of failing to identify younger patient with an increased risk for more severe AKI.

In the SKS cohort the primary renal diagnoses included in the model are those with the greatest numbers (over 130 individuals in each) or of particular interest (vasculitis). The other diagnoses are of multiple etiologies including IgA, focal segmental glomerulosclerosis, membranous, haemolytic uraemic syndrome, lupus nephritis, membranoproliferative glomerulonephritis, drug-induced or amyloid. It is reasonable to consider diabetes and diabetic renal disease as separate as diabetic nephropathy may not be the cause of the primary renal disease in all patients with diabetes. In total 13.2% of all diagnoses in the SKS have an unknown primary renal disease. The lower observed percentages of patients with diabtetes (31.6%) or primary renal diagnoses of diabetes (16.4%) than in other studies may reflect the predominantly Caucasian ethnicity (96.2%) within the SKS group.

Previous analyses from the SKS[24] and other cohorts[25] have demonstrated the importance of primary renal disease in progression of CKD. Our analysis suggests that the cause of CKD may also have bearing on the risk for AKI. It is of interest that, in line with evidence that patients with ADPKD have been shown to have more rapid rates of eGFR loss[26,27] than other causes of CKD, here we have demonstrated that they also appear to be at increased risk for severe AKI episodes. It may be that these two findings are mechanistically associated.

A key message from this study is that for each AKI episode suffered by a patient, there is an increasing likelihood that the next AKI will be more severe and be stage 2 or 3 rather than stage 1. Furthermore, these more severe AKI are associated with an escalating risk for progression to RRT. If a second AKI is stage 2 or 3 (rather than stage 1), the patient has a 14 fold increased risk of reaching ESRD. If a third AKI is stage 2 or 3, the risk of reaching ESRD is 28 fold. Whilst speculative we would propose that recurrent episodes of AKI lead to progressive fibrosis or low-grade inflammation of the kidney could be responsible for this progression to ESRD. Episodic AKI appears to be linked to progression of CKD, regardless of the cause of the AKI.”

AKI episodes were not associated with an increased risk for long-term mortality. This is in contrast with the findings of previous studies.[13,28–30] This difference may relate to the fact that previous investigations have considered patients with more preserved baseline levels of renal function. The uraemic milieu of advanced CKD is a potent competing risk factor that may attenuate the mortality risk otherwise associated with AKI.

### Limitations

This study has limitations that should be carefully considered. Although care was taken to validate AKI events, the aetiology of each AKI episode was not considered. It is therefore likely that different causes of AKI will have different natural histories, pathogenic effects within the kidneys and systemic outcomes. AKI was defined by the KDIGO definition through analysis of serial serum creatinine measurements without corroborative information on urine output. The AKI episodes were retrospectively identified and not totally in hospitalization and therefore the definition of AKI by KDIGO guideline is not fulfilled of the 7-day period limit. It is difficult to distinguish between incomplete renal recovery and a second AKI within short periods of time due to the timing of blood tests in this observational study. A large proportion of acute and chronic renal patients are followed up in an outpatient setting in renal services in the UK and therefore the majority of blood tests in this study originate from outpatients. Whilst this may provide additional data on baseline renal function, it is possible to miss AKI episodes or recovery due to infrequent monitoring or attendance. It is difficult to mitigate for this as more regular blood tests without clear indication may not yield useful information and bring attendant costs and additional procedures. Further research perhaps with the advent of point of care testing may help the renal community develop greater understanding of this peri-AKI period and develop more detailed guidance.

It is also possible that patients who live furthest from our Hospital site may have admissions to other hospitals for AKI management and follow up but for which we did not capture laboratory data. This is likely to explain the association between geographical location and AKI risk. We have also assumed linearity in the rate of decline in renal function. This assumption is not always correct, especially in the period preceding initiation of RRT. [6,31]

Due to the referred nature of the study population, in a hub and spoke model of delivery of renal specialist care, almost 20% of the population had an immune mediated cause for their primary kidney disease (i.e. glomerulonephritis or vasculitis). Although patients with a background of for example ANCA-associated vasculitis can suffer pre-renal AKI, there is also the possibility for flares of intrinsic renal disease to occur. It may therefore be that the quantified risk detailed here cannot be directly applied to a general population CKD cohort that are not cared for in a specialist centre, although we expect the same principal of sequentially increasing risk associated with each AKI suffered by a patient to apply.

For the purpose of analysis, we assumed that in patients who had no AKI events, all repeated measurements contributed to the period prior to a first AKI. It is possible that a small number of these latter observations may have belonged to the period one month prior to an, as yet unobserved, AKI. Previous sensitivity analysis, however, suggests that the impact of this would be negligible. Analysis was unable to include baseline CKD at study entry in the statistical model, which could be influential. However the model does point to AKI being a predictor in CKD progression, future AKI and mortality independent of baseline eGFR given that the latter is in the statistical model.

## Supporting information

S1 File Tables A-I.
(DOCX)
