# Supplementary material for: The influence of multiple episodes of acute kidney injury on survival and progression to end stage kidney disease in patients with chronic kidney disease
Source: PLoS One. 2019 Jul 18;14(7):e0219828. doi: 10.1371/journal.pone.0219828 (PMC6638939; doi:10.1371/journal.pone.0219828)
Supplement: S1 File — Tables A-I. (DOCX) [file pone.0219828.s001.docx]

**Supporting information**

Table A. Partial likelihood estimates for the first AKI event. SE: standard error, HR: hazard ratio, CI: confidence interval.

| **Stage 1** | | | | |
| --- | --- | --- | --- | --- |
| **Variable** | **Estimate** | **SE** | **HR (95% CI)** | **p-value** |
| Baseline age | 0.00 | 0.00 | 1.00 (0.99, 1.01) | 0.50 |
| Base hospital (SRFT=1) | 0.84 | 0.11 | 2.31 (1.86, 2.86) | 0.00 |
| Gender (male=1) | -0.09 | 0.12 | 0.92 (0.73, 1.15) | 0.46 |
| Smoking (ex/current=1) | 0.32 | 0.13 | 1.38 (1.08, 1.77) | 0.01 |
| Alcohol (any intake=1) | -0.22 | 0.11 | 0.80 (0.64, 1.00) | 0.05 |
| Diabetes (type I/II=1) | 0.10 | 0.16 | 1.10 (0.81, 1.51) | 0.55 |
| Cardiovascular (yes=1) | 0.17 | 0.14 | 1.19 (0.91, 1.55) | 0.22 |
| PRD (diabetes=1) | 0.44 | 0.20 | 1.55 (1.04, 2.31) | 0.03 |
| PRD (GN=1) | 0.11 | 0.18 | 1.12 (0.78, 1.61) | 0.54 |
| PRD (immune/vasc=1) | 0.64 | 0.26 | 1.90 (1.14, 3.18) | 0.01 |
| PRD (polycystic=1) | -0.03 | 0.29 | 0.97 (0.55, 1.71) | 0.92 |
| PRD (pyelonephritis=1) | -0.12 | 0.26 | 0.89 (0.54, 1.47) | 0.65 |
| PRD (vascular=1) | -0.15 | 0.17 | 0.86 (0.62, 1.19) | 0.37 |
| **Stage 2/3** | | | | |
| **Variable** | **Estimate** | **SE** | **HR (95% CI)** | **p-value** |
| Baseline age | -0.01 | 0.01 | 0.99 (0.98, 1.00) | 0.02 |
| Base hospital (SRFT=1) | -0.19 | 0.13 | 0.83 (0.64, 1.07) | 0.15 |
| Gender (male=1) | 0.08 | 0.13 | 1.09 (0.85, 1.39) | 0.52 |
| Smoking (ex-current=1) | 0.02 | 0.13 | 1.02 (0.80, 1.30) | 0.90 |
| Alcohol (any intake=1) | -0.07 | 0.12 | 0.94 (0.74, 1.18) | 0.58 |
| Diabetes (type I/II=1) | 0.04 | 0.18 | 1.04 (0.73, 1.50) | 0.82 |
| Cardiovascular (yes=1) | 0.02 | 0.17 | 1.02 (0.74, 1.41) | 0.91 |
| PRD (diabetes=1) | 0.43 | 0.23 | 1.53 (0.97, 2.41) | 0.07 |
| PRD (GN=1) | 0.14 | 0.19 | 1.15 (0.79, 1.67) | 0.46 |
| PRD (immune/vasc=1) | -0.27 | 0.38 | 0.76 (0.36, 1.60) | 0.47 |
| PRD (polycystic=1) | 0.80 | 0.22 | 2.23 (1.46, 3.39) | 0.00 |
| PRD (pyelonephritis=1) | -0.25 | 0.28 | 0.78 (0.45, 1.35) | 0.37 |
| PRD (vascular=1) | -0.04 | 0.19 | 0.96 (0.67, 1.40) | 0.84 |

Table B. Partial likelihood estimates for the second AKI event. SE: standard error, HR: hazard ratio, CI: confidence interval.

| **Stage 1** | | | | |
| --- | --- | --- | --- | --- |
| **Variable** | **Estimate** | **SE** | **HR (95% CI)** | **p-value** |
| First AKI (stage 2/3=1) | -0.71 | 0.28 | 0.49 (0.95, 0.85) | 0.01 |
| Baseline age | 0.01 | 0.01 | 1.01 (1.00, 1.02) | 0.19 |
| Base hospital (SRFT=1) | 0.24 | 0.20 | 1.27 (0.85, 1.88) | 0.24 |
| Gender (male=1) | 0.35 | 0.22 | 1.41 (0.92, 2.17) | 0.11 |
| Smoking (ex-current=1) | -0.43 | 0.22 | 0.65 (0.43, 1.00) | 0.05 |
| Alcohol (any intake=1) | 0.05 | 0.20 | 1.05 (0.71, 1.55) | 0.80 |
| Diabetes (type I/II=1) | 0.21 | 0.28 | 1.23 (0.71, 2.13) | 0.45 |
| Cardiovascular (yes=1) | 0.16 | 0.24 | 1.17 (0.73, 1.88) | 0.51 |
| PRD (diabetes=1) | -0.15 | 0.35 | 0.86 (0.43, 1.72) | 0.68 |
| PRD (GN=1) | -0.18 | 0.32 | 0.84 (0.45, 1.55) | 0.57 |
| PRD (immune/vasc=1) | -0.06 | 0.51 | 0.95 (0.35, 2.57) | 0.91 |
| PRD (polycystic=1) | -0.22 | 0.55 | 0.81 (0.28, 2.36) | 0.70 |
| PRD (pyelonephritis=1) | -0.04 | 0.44 | 0.96 (0.41, 2.28) | 0.93 |
| PRD (vascular=1) | -0.37 | 0.29 | 0.69 (0.39, 1.23) | 0.21 |
| **Stage 2/3** | | | | |
| **Variable** | **Estimate** | **SE** | **HR (95% CI)** | **p-value** |
| First AKI (stage 2/3=1) | 0.13 | 0.29 | 1.14 (0.64, 2.02) | 0.66 |
| Baseline age | 0.00 | 0.01 | 1.00 (0.98, 1.02) | 0.80 |
| Base hospital (SRFT=1) | -0.02 | 0.26 | 0.98 (0.59, 1.63) | 0.94 |
| Gender (male=1) | 0.22 | 0.29 | 1.24 (0.70, 2.20) | 0.45 |
| Smoking (ex-current=1) | 0.39 | 0.31 | 1.48 (0.80, 2.74) | 0.21 |
| Alcohol (any intake=1) | 0.15 | 0.26 | 1.16 (0.70, 1.92) | 0.56 |
| Diabetes (type I/II=1) | 0.19 | 0.39 | 1.21 (0.56, 2.60) | 0.63 |
| Cardiovascular (yes=1) | -0.55 | 0.37 | 0.58 (0.28, 1.189) | 0.14 |
| PRD (diabetes=1) | 0.21 | 0.50 | 1.23 (0.46, 3.29) | 0.68 |
| PRD (GN=1) | 0.11 | 0.42 | 1.12 (0.49, 2.53) | 0.79 |
| PRD (immune/vasc=1) | 0.32 | 0.68 | 1.38 (0.36, 5.27) | 0.64 |
| PRD (polycystic=1) | -0.41 | 0.78 | 0.66 (0.15, 3.02) | 0.60 |
| PRD (pyelonephritis=1) | 0.47 | 0.56 | 1.60 (0.54, 4.77) | 0.40 |
| PRD (vascular=1) | 0.24 | 0.39 | 1.27 (0.60, 2.73) | 0.53 |

Table C. Partial likelihood estimates for a third or more AKI events. SE: standard error, HR: hazard ratio, CI: confidence interval.

| **Stage 1** | | | | |
| --- | --- | --- | --- | --- |
| **Variable** | **Estimate** | **SE** | **HR (95% CI)** | **p-value** |
| Second AKI (stage 2/3=1) | -0.33 | 0.38 | 0.72 (0.34, 1.51) | 0.38 |
| Baseline age | 0.00 | 0.01 | 1.00 (0.97, 1.02) | 0.82 |
| Base hospital (SRFT=1) | -0.24 | 0.33 | 0.79 (0.41, 1.52) | 0.48 |
| Gender (male=1) | -0.26 | 0.34 | 0.77 (0.40, 1.49) | 0.44 |
| Smoking (ex-current=1) | -0.21 | 0.33 | 0.81 (0.43, 1.55) | 0.53 |
| Alcohol (any intake=1) | 0.09 | 0.30 | 1.10 (0.61, 1.99) | 0.76 |
| Diabetes (type I/II=1) | -0.54 | 0.43 | 0.59 (0.25, 1.35) | 0.21 |
| Cardiovascular (yes=1) | 0.51 | 0.38 | 1.67 (0.79, 3.51) | 0.18 |
| PRD (diabetes=1) | 0.71 | 0.53 | 2.03 (0.72, 5.68) | 0.18 |
| PRD (GN=1) | 0.21 | 0.51 | 1.23 (0.45, 3.34) | 0.69 |
| PRD (immune/vasc=1) | 0.74 | 0.73 | 2.09 (0.50, 8.67) | 0.31 |
| PRD (polycystic=1) | -0.34 | 1.08 | 0.71 (0.09, 5.92) | 0.75 |
| PRD (pyelonephritis=1) | -0.21 | 0.71 | 0.81 (0.20, 3.25) | 0.77 |
| PRD (vascular=1) | 0.58 | 0.45 | 1.79 (0.74, 4.32) | 0.20 |
| **Stage 2/3** | | | | |
| **Variable** | **Estimate** | **SE** | **HR (95% CI)** | **p-value** |
| Second AKI (stage 2/3=1) | -0.26 | 0.49 | 0.77 (0.30, 2.02) | 0.60 |
| Baseline age | 0.00 | 0.01 | 1.00 (0.97, 1.03) | 0.81 |
| Base hospital (SRFT=1) | 0.88 | 0.48 | 2.40 (0.95, 6.10) | 0.07 |
| Gender (male=1) | 0.13 | 0.46 | 1.14 (0.46, 2.81) | 0.78 |
| Smoking (ex-current=1) | 0.04 | 0.48 | 1.04 (0.41, 2.68) | 0.93 |
| Alcohol (any intake=1) | -0.63 | 0.45 | 0.54 (0.22, 1.30) | 0.17 |
| Diabetes (type I/II=1) | -1.41 | 0.80 | 0.25 (0.05, 1.18) | 0.08 |
| Cardiovascular (yes=1) | -0.50 | 0.66 | 0.61 (0.17, 2.23) | 0.45 |
| PRD (diabetes=1) | 1.17 | 0.93 | 3.21 (0.52, 19.89) | 0.21 |
| PRD (GN=1) | 0.50 | 0.65 | 1.65 (0.46, 5.93 ) | 0.45 |
| PRD (immune/vasc=1) | 0.98 | 1.19 | 2.66 (0.26, 27.28) | 0.41 |
| PRD (polycystic=1) | 0.94 | 0.78 | 2.56 (0.56, 11.76) | 0.23 |
| PRD (pyelonephritis=1) | -0.24 | 0.91 | 0.78 (0.13, 4.70) | 0.79 |
| PRD (vascular=1) | 0.32 | 0.66 | 1.38 (0.38, 5.00) | 0.63 |

Table D. Partial likelihood estimates for death prior to the first AKI event. SE: standard error, HR: hazard ratio, CI: confidence interval.

| **Variable** | **Estimate** | **SE** | **HR (95% CI)** | **p-value** |
| --- | --- | --- | --- | --- |
| Baseline age | 0.08 | 0.01 | 1.08 (1.07, 1.09) | 0.00 |
| Base hospital (SRFT=1) | -0.51 | 0.12 | 0.60 (0.48, 0.72) | 0.00 |
| Gender (male=1) | 0.24 | 0.12 | 1.27 (1.01, 1.59) | 0.04 |
| Smoking (ex-current=1) | 0.33 | 0.12 | 1.39 (1.09, 1.77) | 0.01 |
| Alcohol (any intake=1) | -0.26 | 0.11 | 0.77 (0.62, 0.95) | 0.02 |
| Diabetes (type I/II=1) | 0.46 | 0.13 | 1.58 (1.23, 2.03) | 0.00 |
| Cardiovascular (yes=1) | 0.31 | 0.11 | 1.36 (1.09, 1.70) | 0.01 |
| PRD (diabetes=1) | -0.03 | 0.19 | 0.97 (0.67, 1.40) | 0.87 |
| PRD (GN=1) | -0.07 | 0.19 | 0.93 (0.64, 1.37) | 0.72 |
| PRD (immune/vasc=1) | -0.56 | 0.46 | 0.57 (0.23, 1.41) | 0.22 |
| PRD (polycystic=1) | -0.27 | 0.35 | 0.77 (0.38, 1.53) | 0.45 |
| PRD (pyelonephritis=1) | -0.14 | 0.27 | 0.87 (0.51, 1.47) | 0.61 |
| PRD (vascular=1) | 0.08 | 0.14 | 1.08 (0.83, 1.42) | 0.56 |

Table E. Partial likelihood estimates for death prior to the second AKI event SE: standard error, HR: hazard ratio, CI: confidence interval.

| **Variable** | **Estimate** | **SE** | **HR (95% CI)** | **p-value** |
| --- | --- | --- | --- | --- |
| First AKI (stage 2/3=1) | 0.32 | 0.23 | 1.38 (0.88, 2.16) | 0.16 |
| Baseline age | 0.05 | 0.01 | 1.05 (1.03, 1.07) | 0.00 |
| Base hospital (SRFT=1) | -0.14 | 0.21 | 0.87 (0.57, 1.31) | 0.50 |
| Gender (male=1) | 0.02 | 0.22 | 1.02 (0.67, 1.57) | 0.91 |
| Smoking (ex-current=1) | 0.22 | 0.26 | 1.25 (0.76, 2.06) | 0.39 |
| Alcohol (any intake=1) | -0.31 | 0.21 | 0.73 (0.49, 1.10) | 0.14 |
| Diabetes (type I/II=1) | -0.17 | 0.28 | 0.85 (0.49, 1.45) | 0.54 |
| Cardiovascular (yes=1) | 0.42 | 0.22 | 1.52 (1.00, 2.32) | 0.05 |
| PRD (diabetes=1) | 0.76 | 0.39 | 2.14 (0.99, 4.62) | 0.05 |
| PRD (GN=1) | 0.17 | 0.39 | 1.18 (0.55, 2.54) | 0.67 |
| PRD (immune/vasc=1) | 1.23 | 0.50 | 3.41 (1.29, 9.03) | 0.01 |
| PRD (polycystic=1) | -0.18 | 0.76 | 0.84 (0.19, 3.68) | 0.81 |
| PRD (pyelonephritis=1) | 0.15 | 0.64 | 1.16 (0.34, 4.04) | 0.81 |
| PRD (vascular=1) | 0.70 | 0.30 | 2.10 (1.12, 3.61) | 0.02 |

Table F. Partial likelihood estimates for death prior to the third AKI event. SE: standard error, HR: hazard ratio, CI: confidence interval.

| **Variable** | **Estimate** | **SE** | **HR (95% CI)** | **p-value** |
| --- | --- | --- | --- | --- |
| Second AKI (stage 2/3=1) | -0.38 | 0.42 | 0.69 (0.30, 1.55) | 0.37 |
| Baseline age | 0.06 | 0.02 | 1.06 (1.02, 1.10) | 0.00 |
| Base hospital (SRFT=1) | 0.15 | 0.43 | 1.16 (0.50, 2.70) | 0.74 |
| Gender (male=1) | -0.64 | 0.38 | 0.53 (0.25, 1.10) | 0.09 |
| Smoking (ex-current=1) | 0.03 | 0.40 | 1.03 (0.47, 2.27) | 0.94 |
| Alcohol (any intake=1) | 0.83 | 0.38 | 2.28 (1.08, 4.84) | 0.03 |
| Diabetes (type I/II=1) | -0.17 | 0.47 | 0.85 (0.34, 2.12) | 0.73 |
| Cardiovascular (yes=1) | 1.03 | 0.41 | 2.80 (1.27, 6.20) | 0.01 |
| PRD (diabetes=1) | 1.07 | 0.59 | 2.90 (0.91, 9.22) | 0.07 |
| PRD (GN=1) | 1.27 | 0.60 | 3.56 (1.10, 11.54) | 0.03 |
| PRD (immune/vasc=1) | 0.53 | 1.15 | 1.69 (0.18, 16.13) | 0.65 |
| PRD (vascular=1) | 0.92 | 0.50 | 2.52 (0.95, 6.69) | 0.06 |

Table G. Partial likelihood estimates for RRT prior to the first AKI event. SE: standard error, HR: hazard ratio, CI: confidence interval.

| **Variable** | **Estimate** | **SE** | **HR (95% CI)** | **p-value** |
| --- | --- | --- | --- | --- |
| Baseline age | -0.03 | 0.01 | 0.97 (0.95, 0.99) | 0.01 |
| Base hospital (SRFT=1) | -0.80 | 0.39 | 0.45 (0.21, 0.96) | 0.04 |
| Gender (male=1) | -0.02 | 0.30 | 0.98 (0.54, 1.78) | 0.94 |
| Smoking (ex-current=1) | 0.61 | 0.33 | 1.84 (0.97, 3.47) | 0.06 |
| Alcohol (any intake=1) | -0.18 | 0.29 | 0.83 (0.47, 1.48) | 0.54 |
| Diabetes (type I/II=1) | -0.27 | 0.55 | 0.76 (0.26, 2.22) | 0.62 |
| Cardiovascular (yes=1) | -1.09 | 0.61 | 0.34 (0.10, 1.12) | 0.08 |
| PRD (diabetes=1) | 1.17 | 0.67 | 3.21 (0.87, 11.81) | 0.08 |
| PRD (GN=1) | 0.16 | 0.51 | 1.17 (0.43, 3.17) | 0.76 |
| PRD (immune/vasc=1) | 0.05 | 0.81 | 1.06 (0.22, 5.16) | 0.95 |
| PRD (polycystic=1) | 0.87 | 0.57 | 2.39 (0.79, 7.26) | 0.12 |
| PRD (pyelonephritis=1) | -0.24 | 0.71 | 0.79 (0.20, 3.16) | 0.74 |
| PRD (vascular=1) | 0.74 | 0.49 | 2.10 (0.80, 5.51) | 0.13 |

Table H. Partial likelihood estimates for RRT prior to the second AKI event. SE: standard error, HR: hazard ratio, CI: confidence interval.

| **Variable** | **Estimate** | **SE** | **HR (95% CI)** | **p-value** |
| --- | --- | --- | --- | --- |
| First AKI (stage 2/3=1) | 2.67 | 0.21 | 14.46 (9.56, 21.87) | 0.00 |
| Baseline age | -0.01 | 0.01 | 0.99 (0.98, 1.00) | 0.01 |
| Base hospital (SRFT=1) | -0.83 | 0.18 | 0.44 (0.31 0.62) | 0.00 |
| Gender (male=1) | 0.24 | 0.15 | 1.27 (0.95, 1.70) | 0.10 |
| Smoking (ex-current=1) | 0.17 | 0.15 | 1.19 (0.90, 1.58) | 0.23 |
| Alcohol (any intake=1) | -0.03 | 0.14 | 0.97 (0.74, 1.27) | 0.82 |
| Diabetes (type I/II=1) | 0.21 | 0.24 | 1.23 (0.77, 1.97) | 0.39 |
| Cardiovascular (yes=1) | -0.16 | 0.21 | 0.85 (0.57, 1.28) | 0.44 |
| PRD (diabetes=1) | 0.20 | 0.28 | 1.22 (0.70, 2.13) | 0.49 |
| PRD (GN=1) | 0.13 | 0.22 | 1.14 (0.74, 1.76) | 0.56 |
| PRD (immune/vasc=1) | 0.32 | 0.42 | 1.38 (0.61, 3.14) | 0.44 |
| PRD (polycystic=1) | 0.75 | 0.23 | 2.11 (1.33, 3.33) | 0.00 |
| PRD (pyelonephritis=1) | 0.83 | 0.29 | 2.29 (1.28, 4.07) | 0.01 |
| PRD (vascular=1) | 0.11 | 0.23 | 1.12 (0.71, 1.77) | 0.63 |

Table I. Partial likelihood estimates for RRT prior to the third AKI event. SE: standard error, HR: hazard ratio, CI: confidence interval.

| **Variable** | **Estimate** | **SE** | **HR (95% CI)** | **p-value** |
| --- | --- | --- | --- | --- |
| Second AKI (stage 2/3=1) | 3.35 | 0.55 | 28.39 (9.71, 83.00 ) | 0.00 |
| Baseline age | -0.03 | 0.01 | 0.98 (0.95, 1.00) | 0.06 |
| Base hospital (SRFT=1) | -1.08 | 0.39 | 0.34 (0.16, 0.73) | 0.01 |
| Gender (male=1) | 0.02 | 0.43 | 1.02 (0.44, 2.39) | 0.96 |
| Smoking (ex-current=1) | 0.18 | 0.39 | 1.20 (0.56, 2.57) | 0.64 |
| Alcohol (any intake=1) | 0.07 | 0.39 | 1.07 (0.50, 2.30) | 0.85 |
| Diabetes (type I/II=1) | -1.50 | 0.70 | 0.22 (0.06, 0.89) | 0.03 |
| Cardiovascular (yes=1) | 0.76 | 0.58 | 2.14 (0.68, 6.72) | 0.19 |
| PRD (diabetes=1) | 1.01 | 0.88 | 2.74 (0.49, 15.43) | 0.25 |
| PRD (GN=1) | 0.49 | 0.60 | 1.63 (0.51, 5.22) | 0.42 |
| PRD (immune/vasc=1) | 0.55 | 0.88 | 1.73 (0.31, 9.73) | 0.54 |
| PRD (polycystic=1) | -0.14 | 0.86 | 0.87 (0.16, 4.65) | 0.87 |
| PRD (pyelonephritis=1) | 0.27 | 0.80 | 1.30 (0.27, 6.30) | 0.74 |
| PRD (vascular=1) | 0.27 | 0.54 | 1.31 (0.46, 3.79) | 0.61 |
